# Elimination of Metastatic Melanoma Using Gold Nanoshell-Enabled Photothermal Therapy and Adoptive T Cell Transfer

**DOI:** 10.1371/journal.pone.0069073

**Published:** 2013-07-23

**Authors:** Adham S. Bear, Laura C. Kennedy, Joseph K. Young, Serena K. Perna, Joao Paulo Mattos Almeida, Adam Y. Lin, Phillip C. Eckels, Rebekah A. Drezek, Aaron E. Foster

**Affiliations:** 1 Center for Cell and Gene Therapy, Baylor College of Medicine, The Methodist Hospital and Texas Children's Hospital, Houston, Texas, United States of America; 2 Interdepartmental Program in Translational Biology and Molecular Medicine, Baylor College of Medicine, Houston, Texas, United States of America; 3 Department of Bioengineering, Rice University, Houston, Texas, United States of America; 4 Department of Electrical and Computer Engineering, Rice University, Houston, Texas, United States of America; 5 Bellicum Pharmaceuticals, Houston, Texas, United States of America; University of Pécs Medical School, Hungary

## Abstract

Ablative treatments such as photothermal therapy (PTT) are attractive anticancer strategies because they debulk accessible tumor sites while simultaneously priming antitumor immune responses. However, the immune response following thermal ablation is often insufficient to treat metastatic disease. Here we demonstrate that PTT induces the expression of proinflammatory cytokines and chemokines and promotes the maturation of dendritic cells within tumor-draining lymph nodes, thereby priming antitumor T cell responses. Unexpectedly, however, these immunomodulatory effects were not beneficial to overall antitumor immunity. We found that PTT promoted the infiltration of secondary tumor sites by CD11b^+^Ly-6G/C^+^ myeloid-derived suppressor cells, consequently failing to slow the growth of poorly immunogenic B16-F10 tumors and enhancing the growth of distant lung metastases. To exploit the beneficial effects of PTT activity against local tumors and on antitumor immunity whilst avoiding the adverse consequences, we adoptively transferred gp100-specific pmel T cells following PTT. The combination of local control by PTT and systemic antitumor immune reactivity provided by adoptively transferred T cells prevented primary tumor recurrence post-ablation, inhibited tumor growth at distant sites, and abrogated the outgrowth of lung metastases. Hence, the combination of PTT and systemic immunotherapy prevented the adverse effects of PTT on metastatic tumor growth and optimized overall tumor control.

## Introduction

*In situ* tumor ablative strategies, including radiofrequency ablation and cryoablation, are effective at destroying localized disease and may stimulate the host immune system to recognize and eliminate remaining tumor cells [1]–[4]. Tumor ablation induces necrotic and apoptotic tumor cell death by direct cytotoxicity and destruction of the tumor microvasculature [5]. Because dying tumor cells provide a source of tumor antigens and induce the expression of natural immune adjuvants, like heat shock proteins [6]–[9] and alarmins [10], they initiate an inflammatory cascade that can promote dendritic cell maturation [11], [12] and culminate in the priming of tumor-specific T cells [13]–[15]. It has been hoped that this immune response would then have secondary beneficial effects on metastatic disease, progression of which is the most common cause of cancer-related deaths.

Unfortunately, few local therapies have led to disease eradication by any immune response they putatively induce. We, therefore, examined in greater detail the immune sequelae induced in the wake of local tumor ablation using hyperthermia with the goal of harnessing stimulatory immune components that could be exploited for the eradication of metastatic disease. We characterized the inflammatory and antitumor immune response to B16-F10 melanoma induced by gold nanoshell-enabled photothermal therapy (PTT), an ablation strategy that utilizes optically tuned gold nanoshells that generate heat upon exposure to near infrared radiation [16], [17]. To evaluate the antitumor effects initiated by PTT, we assessed the growth of distant tumor metastases following primary tumor ablation and identified both stimulatory and inhibitory immune components induced by PTT that promote or suppress immune responses. To enhance systemic antitumor effects, we determined if the immunostimulatory environment induced by PTT could be exploited to promote the expansion and function of adoptively transferred tumor-specific T cells.

We found that PTT promoted the expression of proinflammatory cytokines and chemokines and induced the maturation of dendritic cells (DC) within tumor-draining lymph nodes. These effects did indeed lead to the priming of antitumor CD8^+^ effector T cell responses. Unfortunately, this response also promoted the generation of myeloid-derived suppressor cells and subsequently enhanced metastatic tumor growth. The effects of PTT were, however, sufficient to promote the expansion and function of adoptively transferred tumor-specific T cells, such that the combination of PTT and adoptive T cell therapy (ATCT), but not either component alone, benefited both local and metastatic disease. These data suggest that *in situ* tumor ablation and adoptive immunotherapy can act in a complementary fashion and may be of value for treatment of human cancer.

## Materials and Methods

### Mice

C57BL/6J, Albino C57BL/6J-Tyr-2J/J, and B6.Cg-Thy1a/Cy Tg(TcraTcrb)8Rest/J [18] mice were purchased from Jackson Laboratories (Bar Harbor, ME) and maintained in a pathogen-free mouse facility at Baylor College of Medicine according to institutional guidelines. This study was carried out in strict accordance with the recommendations of the Guide for the Care and Use of Laboratory Animals of the National Institutes of Health. This study was approved by the Institutional Animal Care and Use Committees of Baylor College of Medicine. All procedures were performed under anesthesia, and strong efforts were made to minimize animal suffering.

### Cell lines

The B16-F10 melanoma cell line (H-2k^b^) was obtained from the American Type Culture Collection and used within 6 months of receipt. ATCC utilizes COI for interspecies identification and STR analysis for intraspecies identification. The B16-OVA cell line was kindly provided by Dr. Xiao-Tong Song at Baylor College of Medicine as previously described [19]. All cell lines were screened and tested negative for *Mycoplasm*a contamination by PCR analysis. B16 melanoma tumor cells were cultured in HyClone RPMI 1640 (Logan, UT) supplemented with 10% fetal calf serum (FCS) and 2 mM glutamax (Invitrogen, Carlsbad, CA) at 37°C and 5% CO_2_. B16-OVA media was supplemented with 0.5 mg/mL G418.

### Mouse tumor model

B16-F10 and B16-OVA tumors were established on the lower flanks of C57BL/6J mice by the subcutaneous injection of 5×10^5^ tumor cells. To establish lung metastases, 5×10^5^ B16-F10 tumor cells were administered intravenously via tail vein infusion. The length and width of tumors were measured 2–3 times per week by digital caliper and volumes were calculated using the formula (length^2^ × width ×0.5236) as previously described [20].

### Gold nanoshell synthesis

Hollow gold nanoshells, tuned to the NIR, were synthesized via a galvanic reaction in which gold tetrachloroauric acid (HAuCl_4_) was reduced onto silver nanoparticles serving as sacrificial templates. The process was adapted and modified from Prevo *et al*. [21]. All chemicals were purchased from Sigma-Aldrich. In a typical experiment, synthesis of hollow gold nanoshells were prepared by first aging an aqueous solution of 0.2 mM silver nitrate (AgNO_3_) in the presence of 0.5 mM sodium citrate. After aging the solution, 50 mL aliquots were raised to ∼60°C and a 1 mL injection of 100 mM sodium borohydride (NaBH_4_) solution was added. The solution was allowed to stir for a minimum of 1 hour. After cooling to room temperature, additional AgNO_3_ was added to grow the cores by first injecting 1 mL of 200 mM hydroxy1amine hydrochloride (H_3_NO) to the solution followed by an injection of 200 µL of 0.1 M AgNO_3_. After aging the solution, hollow gold nanoshells were tuned to ∼800 nm wavelength by adding appropriate amounts of 1% HAuCl_4_ to the silver nanoparticle solution and the silver cores were etched via galvanic replacement. The resultant hollow gold nanoshells were quadruple washed via centrifuge in distilled water. All reagents and byproducts were removed from the solution.

### Gold nanoshell-enabled photothermal therapy

Nanoparticles were sterilized by passing the solution through a 0.22 µm polyethylsulfone syringe filter. The day prior to PTT, particles were coated with sterile polyethylene glycol (PEG-SH  = 5 kDa) by adding an optimized amount of 1 mM PEG solution. The optimized ratio of PEG molecules to gold nanoparticles was determined using a salt stability assay. The nanoparticles and PEG were incubated overnight at 4°C. Excess PEG molecules were then removed by centrifuging the nanoparticles and removing the supernatant. The nanoparticles were then resuspended in sterile 1× PBS immediately prior to injection.

Tumors were treated with PTT once they reached a size of 5–8 mm in diameter (day 10–14). The tumor region was first shaved, then directly injected with 20 microliters of PEGylated hollow gold nanoshells (40–42 nm outer diameter, shell thickness  = 5–7 nm, OD 40). Nanoshells were allowed to disperse throughout the tumor for 5 minutes. The tumor was then swabbed with 100% glycerol. A near-infrared laser (Coherent diode array laser, λ = 808 nm, 3 W/cm^2^, spot diameter  = 8 mm) irradiated the tumor for 3 minutes.

### Cytokine and chemokine multiplex

Serum was isolated from PTT-treated mice 24 hours and 96 hours post-ablation. As controls, serum was also obtained from untreated, tumor-bearing mice. Serum cytokines and chemokines were analyzed using a 32-plex murine cytokine/chemokine array obtained from Millipore (Billerica, MA) as per the manufacturer's instructions.

### Antibodies and flow cytometric analysis

Antibodies to CD4, CD8, CD40, CD80, CD86, I-A/I-E, CD11c, CD11b, and Ly-6G/C were purchased from BD Pharmingen (San Jose, CA). Intracellular FoxP3 staining was performed using a Mouse Regulatory T Cell Staining Kit obtained from eBioscience (San Diego, CA) as per the manufacturer's instructions. Stained cells were analyzed on a FACSCalibur instrument (BD, Becton Dickinson, Mountain View, CA) and analyzed using CellQuest software (BD).

### *In vivo* DC maturation

Subcutaneous B16-F10 tumor cells were established on the lower flanks of mice. Tumors were treated with PTT once they reached a size of 5–8 mm in diameter. Forty-eight hours post-PTT, mice were euthanized and tumor-draining (inguinal) lymph nodes were harvested. Lymph nodes were homogenized through a 70 μm filter to create a single cell suspension then stained with antibodies to CD11c, CD40 CD80, CD86, and I-A/I-E. DC phenotype was assessed by flow cytometry after gating on viable CD11c^+^ cells.

### Analysis of contralateral tumor microenvironment

Contralateral tumors were isolated from mice 9 days following PTT of primary day 14 established B16-OVA tumors. Tumors were homogenized through a 70 μm filter to create a single cell suspension. T cell subsets were stained using antibodies to CD4, CD8, and FoxP3 and analyzed by flow cytometry after gating on viable cells within the lymphocyte region. Myeloid-derived suppressor cells were stained using antibodies to CD11b and Ly-6G/C after gating on viable cells.

### Isolation of myeloid-derived suppressor cells following PTT

B16-F10 tumors were established on opposing flanks on day 0. Primary tumors were ablated by PTT on day 7. The control group consisted of untreated tumor-bearing mice. Mice were euthanized on day 12 for analysis. Myeloid-derived suppressor cells (MDSC) were isolated from the spleens of mice by magnetic separation of Gr-1^High^Ly6G^+^ cells using a Myeloid-Derived Suppressor Cell Isolation Kit purchased from Miltenyi Biotec (Cologne, Germany) per manufacturer's protocol.

### Adoptive T cell transfer

Lymphocytes were isolated from the spleens of pmel mice by density gradient separation using Lympholyte-M solution (CEDARLANE Laboratories, Burlington, NC). Lymphocytes were maintained in Hyclone RPMI 1640 supplemented with 10% FCS, 2 mM glutamax, 10 mM HEPES, and 50 μM β-mercaptoethanol at 37°C and 5% CO_2_. T cells were activated using 5 μg/mL concanavalin A (Sigma, St. Louis, MO) and expanded using 10 ng/mL murine recombinant IL-2 (R&D Systems, Minneapolis, MN) for 10–14 days. Twenty-four hours following PTT, pmel T cells were washed, resuspended in PBS and infused intravenously via tail vein (1×10^7^ cells/mouse).

### Thymidine incorporation assay

Spleens were harvested from C57BL/6J mice and dissociated. RBC lysis was performed on spleens using RBC lysis buffer (BD Biosciences; Franklin Lakes, NJ). Splenocytes were seeded in triplicate into 96-well round bottom plates at 1×10^5^ cells per well in complete RPMI containing 10 ng/mL murine recombinant IL-2 following CD3/CD28 activation of T cells using Mouse T-Activator Dynabeads (Invitrogen). Cells were cultured in the absence or presence of Gr-1^High^Ly6G^+^ cells at the following T cell: MDSC ratios: 1∶0, 1∶1, 1∶0.5 and 1∶0.25. T cells were pulsed with 5 μCi [^3^H] thymidine (Amersham Pharmacia Biotech, Piscataway, NJ) 72 hours after stimulation for 18 hours. The cells were then harvested onto glass filter strips and analyzed using a TriCarb 2500 RT β-counter (Packard Biosciences, Downers Grove, IL).

### IFN-γ ELISpot assay

The enzyme-linked immunospot (ELISpot) assay was performed as previously described [22]. Spleens and/or tumor-draining lymph nodes were harvested and dissociated. RBC lysis was performed on spleens using RBC lysis buffer (BD Biosciences; Franklin Lakes, NJ). T cells were activated for 24 hours and IFN-γ secretion was detected using antibodies from Mabtech (Nacka, Sweden). The number of spot forming cells (SFCs) was quantified in a blinded manner (Zellnet Consulting, New York, NY).

To detect immune priming post-PTT, CD8^+^ T cells were purified from spleens by magnetic bead selection using CD8^+^ (Ly-2) microbeads purchases from Miltenyi Biotec and syngeneic DC were used as antigen presenting cells. Bone marrow-derived DC were generated using IL-4 and GM-CSF as previously described [23]. DC were pulsed with tumor lysate for 24 hours then matured for an additional 24 hours using 50 ng/mL LPS (Sigma). 5×10^5^ CD8^+^ T cells were plated in triplicate in the presence of 2×10^4^ DC.

To measure the suppressive function of MDSC following PTT, 1×10^5^ splenocytes were plated in triplicate following CD3/CD28 activation of T cells using Mouse T-Activator Dynabeads (Invitrogen). Cells were cultured in the absence or presence of Gr-1^High^Ly6G^+^ cells at the following T cell: MDSC ratios: 1∶0, 1∶1, 1∶0.5 and 1∶0.25.

To evaluate the pmel T cell response following ATCT, 1×10^6^ splenocytes or TDLN cells were plated in triplicate in the presence of 10 μg/mL human hgp100 peptide (hgp100_25–33_: KVPRNQDWL) obtained from Genemed Synthesis Inc. (San Antonio, TX).

### Statistical analysis

Student's t-test was used to evaluate differences in the expression of cytokines and chemokines, MFI values, tumor growth in responses to treatment, as well as ELISpot data. For comparisons of more than two groups, we performed Analysis of Variance (ANOVA) followed by Student's t-tests with multiple comparison adjustment. Percentage differences in tumor infiltrating cell types and tumor recurrence as well as fold changes in bioluminescence were analyzed using a z-test for difference in proportion. Significance was set a p<0.05. (*) represents p<0.05, (**) represents p<0.01, and (***) represents p<0.001.

## Results

### PTT stimulates the expression of pro-inflammatory cytokines and chemokines

**Figure 1:**
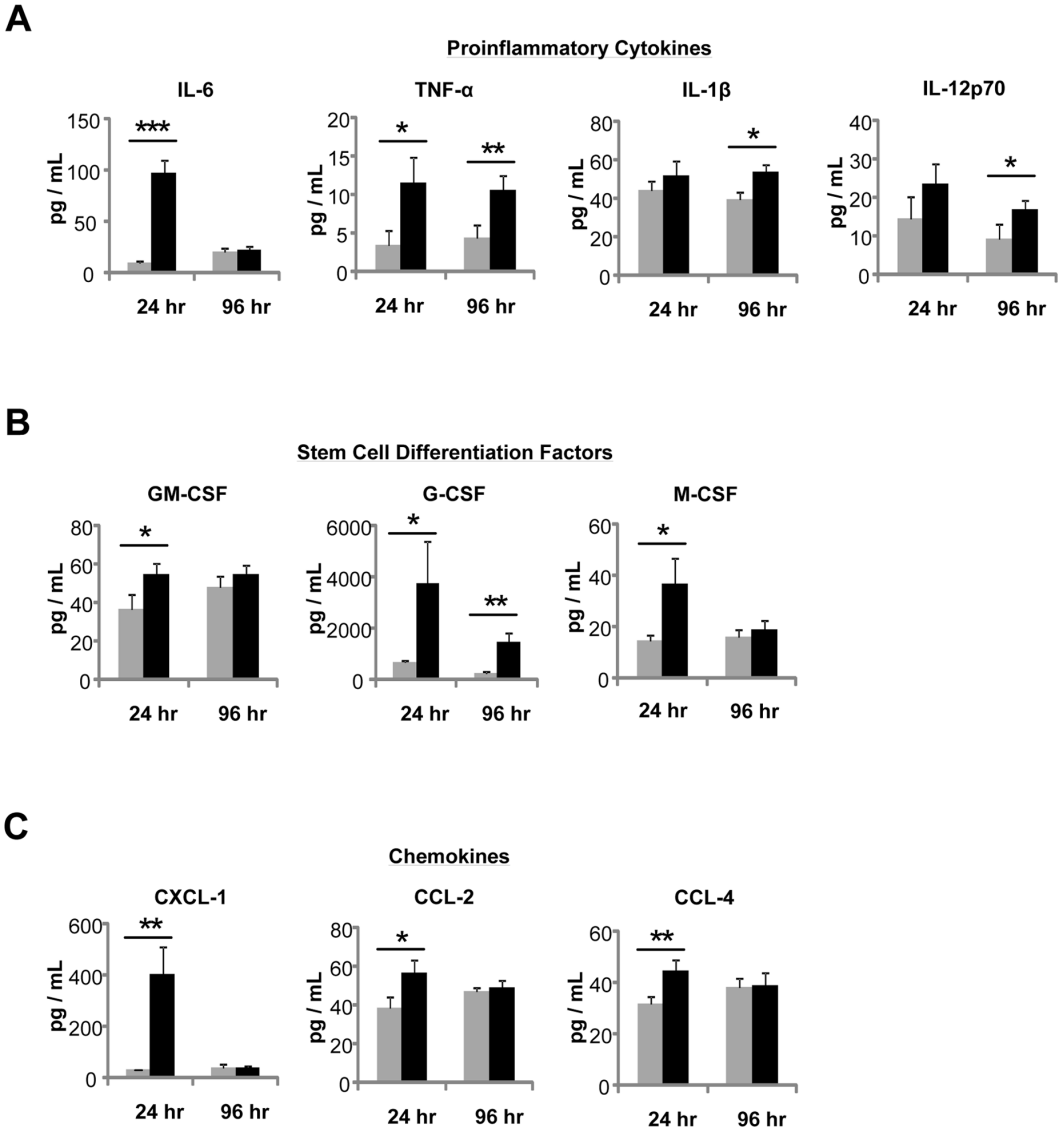
PTT stimulates the expression of proinflammatory cytokines and chemokines. Serum from B16-F10 tumor bearing mice was isolated at 24 hours (n = 10 per group) and 96 hours (n = 8 per group) post-PTT and analyzed using a 32-plex cytokine/chemokine array. PTT induced the expression of the pro-inflammatory cytokines (**A**) IL-6, TNF-α, IL-1β, and IL-12p70, cytokines that induce stem cell differentiation (**B**) GM-CSF, G-CSF, M-CSF, and chemokines (**C**) CXCL-1, CCL-2 and CCL-4. Grey bars represent untreated tumor-bearing mice. Black bars represent PTT-treated mice. Data are presented as the mean +/− SEM. *p<0.05, **p<0.01, ***p<0.001; Student's t-test.

To enable PTT, we synthesized 40 nm hollow GNS (**Figure S1**) tuned to absorb NIR light at ∼808 nm (**Figure S2**). To better understand the immune environment established following PTT, we analyzed inflammatory cytokines and chemokines present in the serum of PTT-treated mice 24 hours and 96 hours post-ablation. PTT induced the expression of the proinflammatory cytokines IL-6, TNF-α, IL-1β, and IL-12p70 (**Figure 1A**). IL-6 was highly expressed at 24 hours and normalized by 96 hours, whereas TNF-α remained elevated for 96 hours. Both IL-1β and IL-12p70 were elevated at 96 hours. PTT also induced cytokines that stimulate stem cell survival, growth, and differentiation (**Figure 1B**). Both GM-CSF and M-CSF were significantly elevated 24 hours following PTT, while G-CSF was expressed at significantly high levels for 96 hours. In addition to cytokines, PTT induced the transient expression of chemokines that direct the migration of immune cells to sites of inflammation (**Figure 1C**). CXCL1, which serves to attract neutrophils to the burn site, was significantly higher 24 hours post-PTT. CCL2 and CCL4, which attract monocytes, macrophages and DC, were also significantly upregulated at the same time point. Overall, PTT elicited a similar immune response to that observed in burn patients, and the proinflammatory cytokine/chemokine profile is consistent with the typical wound healing process [24], [25].

### PTT induces the maturation of DC within tumor-draining lymph nodes

**Figure 2:**
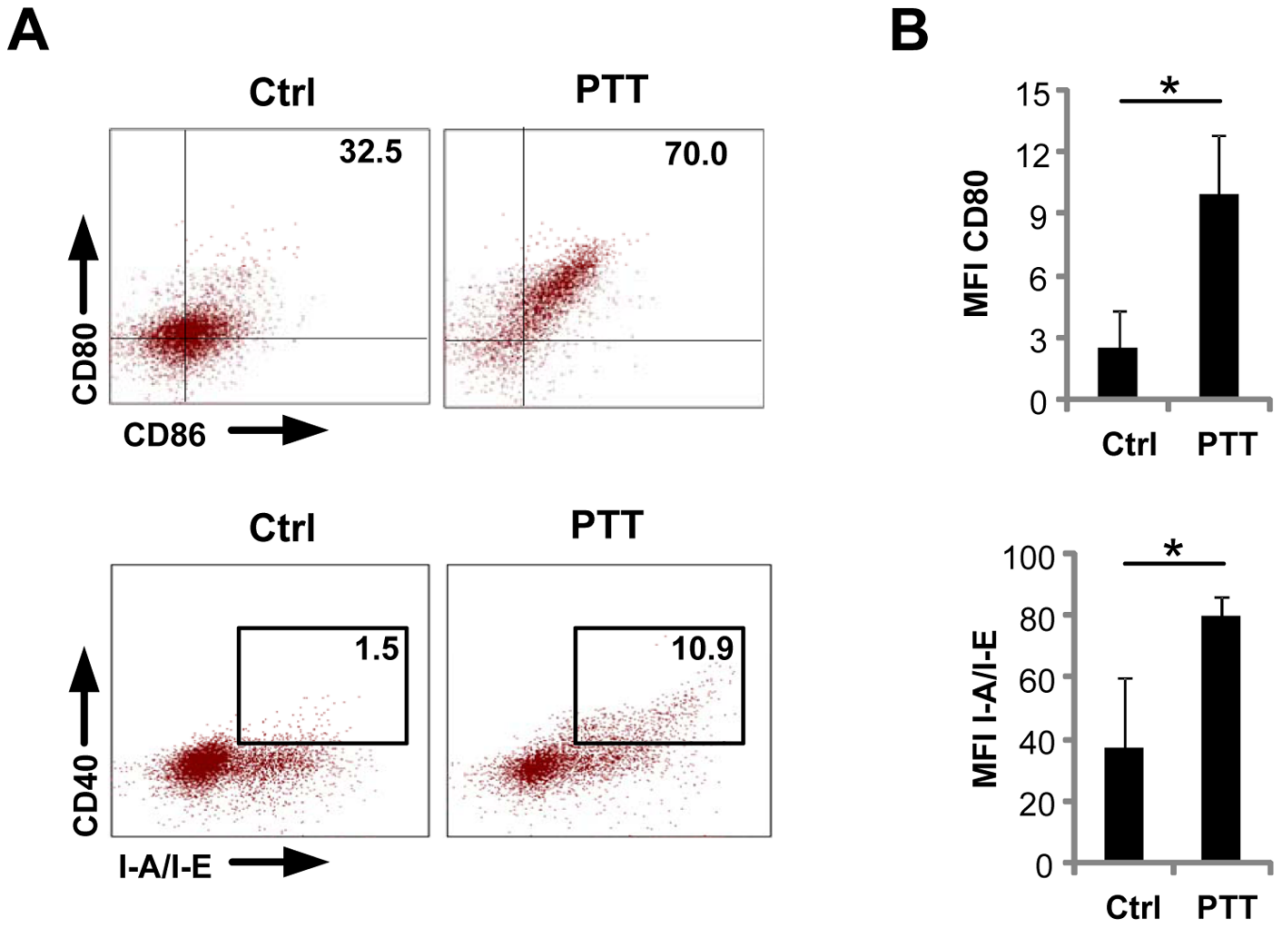
PTT induces the maturation of DC within TDLN. . B16-F10 tumor bearing mice were treated with PTT. 48 hours post-PTT, TDLN were isolated and stained with antibodies to CD11c, CD80, CD86, CD40 and I-A/I-E and analyzed by flow cytometry. DC maturation was assessed by gating on viable CD11c^+^ cells. Untreated tumor-bearing mice were used as controls (n = 3 per group). (**A**) Representative FACS plots of CD80, CD86, CD40 and I-A/I-E expression. (**B**) Corresponding mean fluorescent intensity (MFI) quantification of CD80 and I-A/I-E expression. Data represent the mean +/− SD. *p<0.05; Student's t-test.

To determine if PTT results in the activation of DC subsets, we engrafted mice with B16-F10 tumors, which were then subjected to PTT. Forty-eight hours later, mice were euthanized and the maturation status of DC within the spleen and tumor-draining lymph nodes (TDLN) was assessed using flow cytometry by costaining for CD11c and the maturation markers CD80, CD86, I-A/I-E, and CD40. The maturation status of splenic DC appeared unaffected by PTT (data not shown). However, DC maturation could be detected in TDLN post-PTT (**Figure 2**). These data suggest that PTT initiates a robust proinflammatory process that leads to DC activation within the tumor microenvironment, an effect that could serve to prime antitumor immune responses.

### PTT induces systemic effects that influence T cell and MDSC tumor infiltration

**Figure 3:**
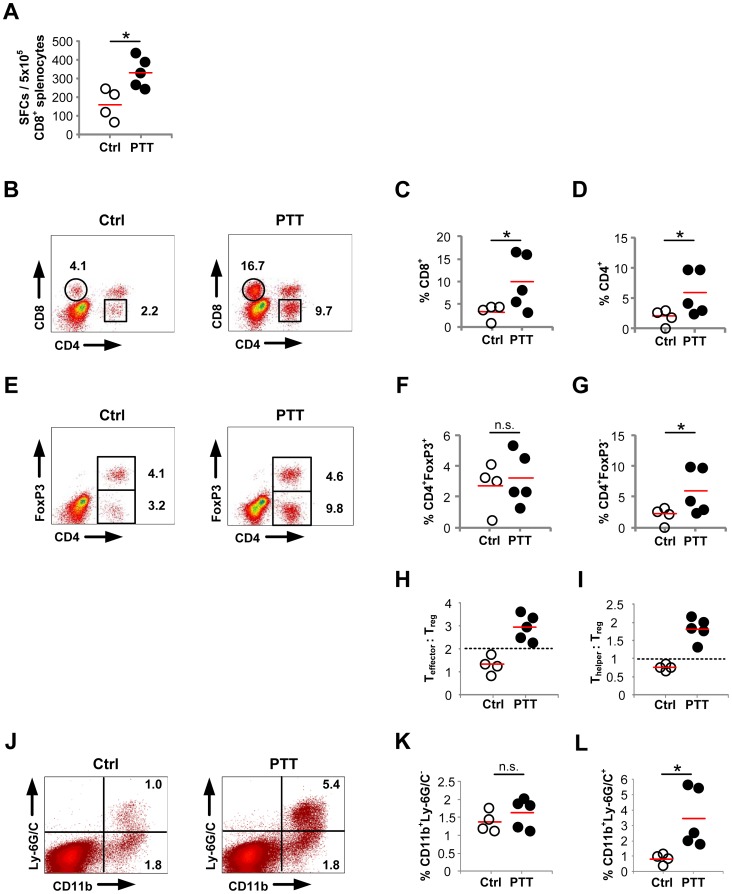
PTT induces systemic effects that influence T cell and MDSC tumor infiltration. B16-OVA tumors were established on opposing flanks on days 0 and 7, respectively. Primary tumors were ablated by PTT on day 14. Mice were euthanized on day 23 for analysis of contralateral tumors. The control group consisted of untreated tumor-bearing mice (Ctrl: n = 4, PTT: n = 5). (**A**) IFN-γ ELISpot assay using CD8^+^ splenocytes stimulated using LPS-matured DC pulsed with tumor-lysate. (**B**) Representative FACS plots and corresponding quantitative data demonstrating (**C**) CD8^+^ and (**D**) CD4^+^ T cells infiltrating contralateral tumor sites. (**E**) Representative FACS plots and corresponding quantitative data demonstrating (**F**) CD4^+^FoxP3^+^ Treg cells and (**G**) CD4^+^FoxP3^−^ Thelper cells within contralateral tumor sites. (**H**) Teffector:Treg and (**I**) Thelper:Treg ratios within contralateral tumors. (**J**) Representative FACS plots and corresponding quantitative data measuring (**K**) CD11b^+^Ly-6G/C^−^ macrophages and (**L**) CD11b^+^Ly-6G/C^+^ MDSC within contralateral tumor sites. Red line represents the mean. The data is representative of two or more experiments. *p<0.05; z-test.

To determine if PTT leads to the priming of tumor-specific effector T cells, CD8^+^ T cells were isolated from the spleens of B16-OVA tumor bearing mice 7 days post-PTT. Their antigen specificity was analyzed by an IFN-γ ELISpot assay following stimulation with tumor lysate-pulsed DC in treated and untreated tumor-bearing mice. PTT-treated mice exhibited significantly greater numbers of IFN-γ secreting cells, indicating PTT induced the priming of antitumor CD8^+^ effector T cell responses (**Figure 3A**).

To analyze the effects of PTT on the immune response to distal tumors, B16-OVA tumors were established on opposing flanks on days 0 and 7, respectively. Primary tumors were ablated by PTT on day 14, and mice were euthanized on day 23 for analysis of contralateral tumors. Tumor infiltrating cells were analyzed by flow cytometry following staining with antibodies to CD8, CD4, and FoxP3. Compared to untreated tumor-bearing mice, PTT-treated mice had significantly increased levels of infiltrating CD8^+^ and CD4^+^ T cells in the contralateral tumor (**Figure 3B–D**). The increase in CD4^+^ cell numbers was due to a rise in the proportion of CD4^+^FoxP3^−^ helper T cells in PTT-treated animals (**Figure 3E and G**), and there was no equivalent rise in CD4^+^FoxP3^+^ regulatory T cells (Tregs) (**Figure 3E and F**). The net result of PTT was an increase of both the Teffector:Treg and Thelper:Treg ratios within the tumor microenvironment (**Figure 3H and I**), which is significant because the efficacy of immunotherapeutic regimens correlates with a shift in the intratumoral balance from Tregs to Teffector cells [26].

Although Tregs were unchanged following PTT, we found a substantial rise in the suppressive myeloid-derived suppressor cell subset (MDSC) within the distal tumor microenvironment. While PTT did not influence the number of CD11b^+^Ly-6G/C^−^ macrophages (**Figure 3J and K**) in contralateral tumors, the levels of CD11b^+^Ly-6G/C^ +^ MDSC were greatly increased in the distal tumor microenvironment (**Figure 3J and L**).

In summary, PTT primes effector and helper T cells that infiltrate contralateral tumor sites. PTT does not increase Treg infiltration of tumors, but promotes distal tumor accumulation of MDSC. We did not detect any correlation between the degree of CD4^+^, CD8^+^, and CD11b^+^Ly-6G/C^+^ cell infiltration following PTT and tumor burden at the time of PTT or euthanasia, which suggests these changes within the tumor microenvironment are a result of PTT and not a direct effect of overall tumor burden.

### PTT induces systemic MDSC expansion capable of suppressing T cell proliferation and function

**Figure 4:**
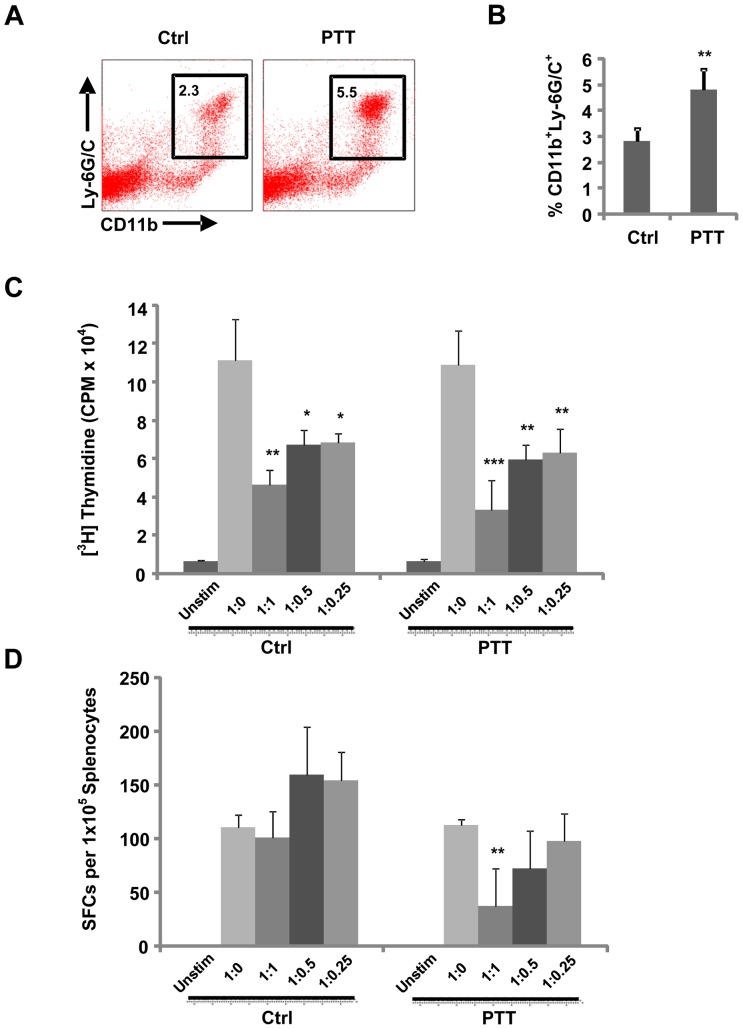
PTT induces systemic MDSC expansion capable of suppressing T cell proliferation and function. B16-F10 tumors were established on the flanks of C57BL/6J mice on day 0. Tumors were ablated by PTT on day 7. Mice were euthanized on day 12 for analysis. The control group consisted of untreated tumor-bearing mice (Ctrl: n = 4, PTT: n = 5). (**A**) Representative FACS plots and (**B**) corresponding quantitative data demonstrating CD11b^+^Ly6G/C^+^ cells within the spleens of control and PTT-treated mice. (**C**) Thymidine incorporation assay to measure T cell proliferation 72 hours following CD3/CD28 stimulation of splenocytes at various ratios of splenocytes to Gr-1^High^Ly-6G^+^ cells (T cell: MDSC). (**D**) IFN-γ secretion to measure T cell function in response to CD3/CD28 stimulation at various ratios of splenocytes to Gr-1^High^Ly-6G^+^ cells (T cell: MDSC). The data is representative of two or more experiments. *p<0.05, **p<0.01, ***p<0.001; ANOVA followed by Student's t-test with multiple comparison adjustment.

CD11b^+^Ly-6G/C^+^ cells comprise a heterogeneous population of myeloid cells and granulocytes, and not all of these cells are immunosuppressive. Therefore, we sought to determine whether PTT induces systemic expansion of this particular cell population and if these cells do in fact induce T cell dysfunction. We treated B16-F10 tumor-bearing mice with PTT and isolated spleens 5 days after treatment. The control group consisted of untreated tumor-bearing mice. Following PTT, we noted a significant expansion of CD11b^+^Ly-6G/C^+^ cells within the spleens of mice in comparison to untreated controls (**Figure 4A and B**). We next evaluated the ability of these cells to induce T cell dysfunction. We isolated Gr-1^High^Ly6G^+^ cells from the spleens of control and PTT-treated mice and measured their effect on T cell expansion and IFN-γ secretion. Utilizing a thymidine incorporation assay, we found that Gr-1^High^Ly6G^+^ cells isolated from both control and PTT-treated mice significantly inhibit T cell proliferation following CD3/CD28 stimulation at T cell: MDSC ratios of 1∶1, 1∶0.5 and 1∶0.25 (**Figure 4C**). Interestingly, we found Gr-1^High^Ly6G^+^ cells isolated only from PTT-treated mice inhibited IFN- γ secretion following CD3/CD28 activation, whereas Gr-1^High^Ly6G^+^ cells isolated from control mice had no effect (**Figure 4D**). These data suggest that PTT induces the systemic expansion of Gr-1^High^Ly6G^+^ MDSC with enhanced suppressive activity in comparison to untreated control mice.

### PTT of a single tumor site can slow the growth of distant pre-established B16-OVA tumors but not less immunogenic B16-F10 tumors

**Figure 5:**
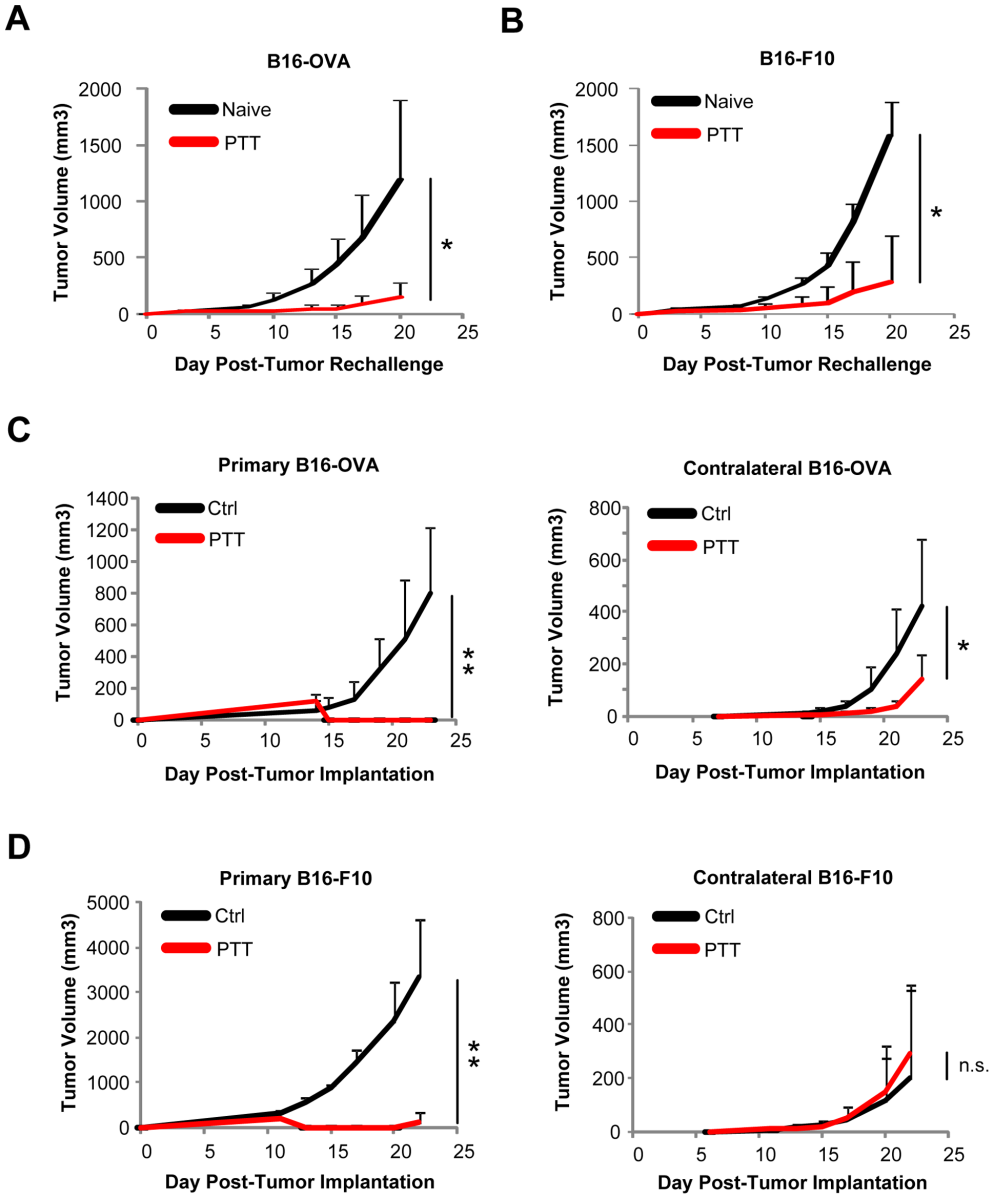
PTT of a single tumor site can slow the growth of distant pre-established B16-OVA tumors but not less immunogenic B16-F10 tumors. (**A**) B16-OVA or (**B**) B16-F10 tumor-bearing mice were treated with PTT then rechallenged with corresponding tumor cells 10 days post-ablation. Naïve mice were used as controls (n = 5 per group). Primary and secondary (**C**) B16-OVA or (**D**) B16-F10 were established on opposing flanks on days 0 and 6, respectively. Primary tumors were ablated with PTT once they reached a size of 5–8 mm (day 12–14). Control mice did not receive PTT (n = 5 per group). Data represent tumor growth over time and is displayed as mean +/− SD. The data is representative of two or more experiments. *p<0.05, **p<0.01; Student's t-test.

Although PTT can treat localized tumors and induce the immunomodulation described above, the combination of an increased effector cell and an increased MDSC response calls into question the overall potency of this antitumor immunity. We first determined if PTT could prime an adaptive antitumor immune response in a tumor rechallenge model targeting either B16-OVA, a highly immunogenic tumor expressing the artificial antigen ovalbumin, or the more weakly immunogenic B16-F10 tumor. These tumors were established on a single flank and then treated with PTT once they reached 5–8 mm. Day 10 post-PTT, mice were rechallenged on the contralateral flank with the corresponding tumor cells and growth was monitored. We found that PTT-treated mice could reject rechallenge with B16-OVA (**Figure 5A**) and with the less immunogenic B16-F10 tumors (**Figure 5B**).

We further tested the potency of the immune response by determining whether the immune response established by PTT at one tumor site could elicit antitumor activity at a pre-established distant tumor site. We engrafted mice with primary B16-OVA or B16-F10 tumors on a single flank. After 6 days, mice were injected with the same tumor on the contralateral flank. When the primary tumors reached 5–8 mm (day 12–14), they were treated with PTT and the subsequent growth of both treated and contralateral tumors was followed. PTT ablated the treated tumors derived from B16-OVA and B16-F10 (**Figure 5C and D**). Growth of the contralateral tumor was also inhibited in mice with B16-OVA tumors (**Figure 5C**), but this distal immune effect did not occur in mice implanted with the less immunogenic B16-F10 tumors (**Figure 5D**).

### PTT promotes the expansion of adoptively transferred pmel T cells

Although PTT can prime adaptive antitumor immune responses, the immune response induced by PTT could not inhibit the growth of distant B16-F10 tumors. This data suggests that to treat metastatic disease the immune response post-PTT must be further modulated by applying combinational therapies that work in concert with PTT. We reasoned that the antitumor T-cell response induced by PTT was insufficient to overcome the growth of tumors, likely because the beneficial effects of the pro-inflammatory environment were countered by an associated rise in MDSC levels, resulting in little benefit to the immunosuppressive tumor environment. We therefore measured the effects of infusing tumor-directed activated T cells to exploit the T cell promoting proinflammatory environment and avoid the concomitant inhibition of T cell activation associated with MDSC.

**Figure 6:**
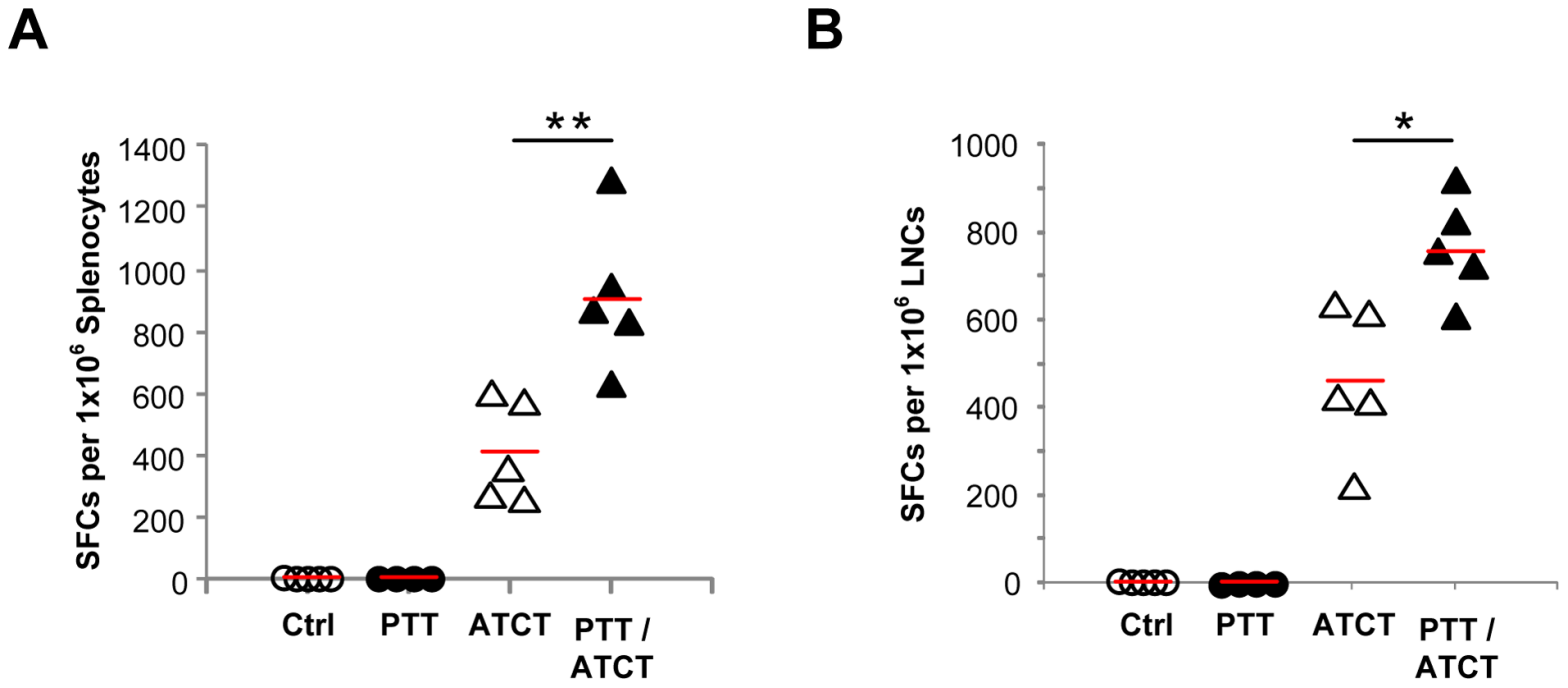
PTT promotes the expansion of adoptively transferred pmel T cells. Primary B16-F10 tumors were established on day 0, and contralateral tumors were established on day 6. Primary tumors were ablated by PTT on day 10 followed by pmel ATCT on day 11. On day 20, mice were euthanized and tissues were harvested for analysis. IFN-γ secretion in response to hgp100 by cells isolated from the (**A**) spleen and (**B**) TDLN. Treatment groups consisted of untreated tumor bearing mice (n = 5), PTT alone (n = 4), ATCT alone (n = 5), and dual PTT/ATCT (n = 5). The data is representative of two or more experiments. *p<0.05, **p<0.01; ANOVA followed by Student's t-test with multiple comparison adjustment.

We first evaluated the potential stimulatory effects of PTT on adoptively transferred pmel T cells, specific for the melanoma tumor antigen gp100, by measuring the expansion and function of pmel T cells infused post-ablation of B16-F10 tumors. Primary and contralateral B16-F10 tumors were established on days 0 and 6 as before. Primary tumors were ablated by PTT on day 10. Twenty-four hours following ablation, pmel T cells were infused i.v. We then performed an ELISpot assay to measure the frequency of pmel T cells within spleens (**Figure 6A**) and tumor-draining lymph nodes (TDLN) (**Figure 6B**) 9 days after adoptive transfer (day 20) by measuring IFN-γ release in response to hgp100. IFN-γ secretion was only detected in conditions administered pmel T cells, and the highest number of tumor-reactive pmel T cells in the spleen (ATCT: 417+/−167, PTT/ATCT: 909+/−240) and TDLN (ATCT: 461+/−167, PTT/ATCT: 761+/−114) were found in mice treated with dual PTT/ATCT. These data suggest that PTT can promote pmel T cell expansion.

### ATCT prevents tumor recurrence post-PTT, while PTT enhances the efficacy of ATCT

**Figure 7:**
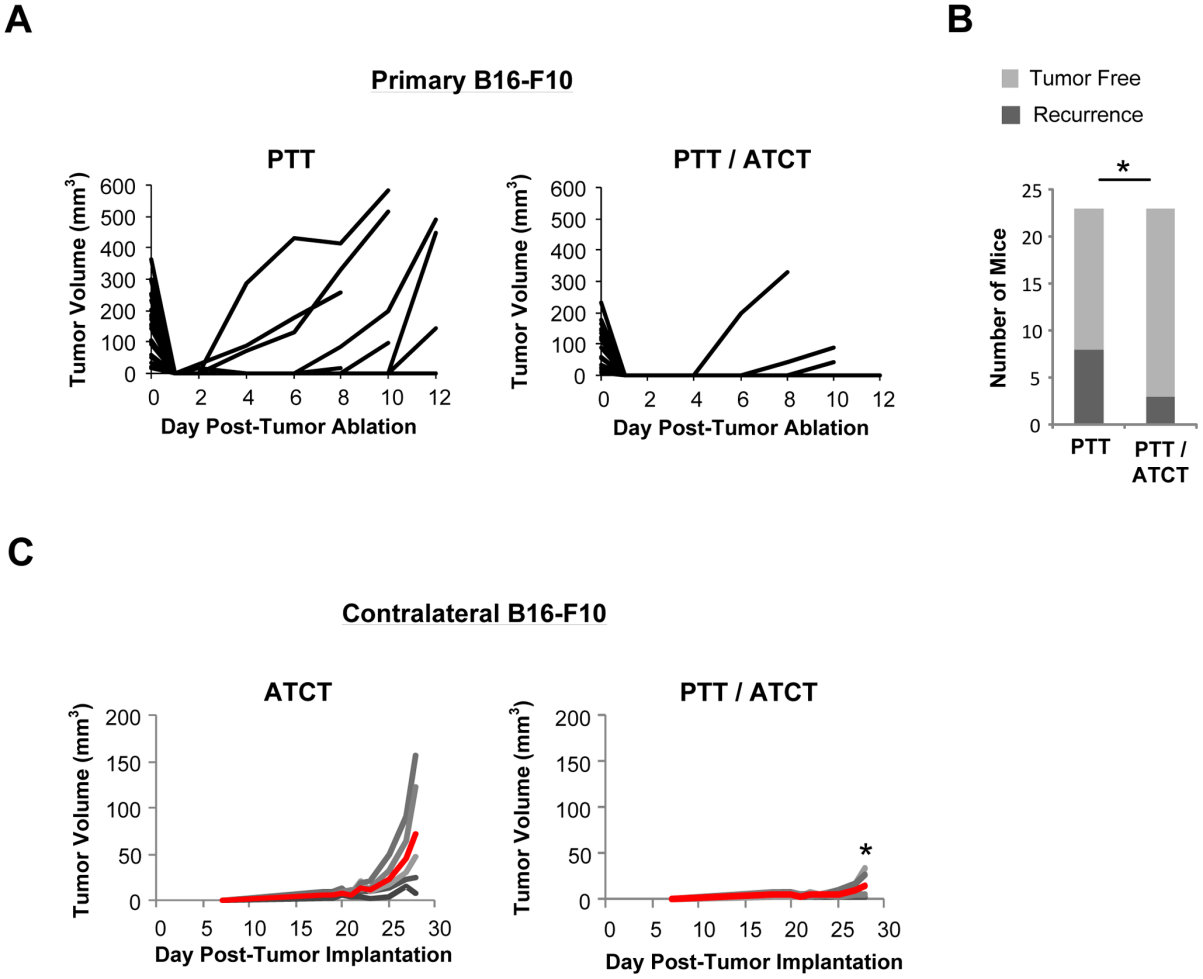
ATCT prevents tumor recurrence post-PTT, while PTT enhances the antitumor efficacy of ATCT. Primary B16-F10 tumors were established on day 0, and contralateral tumors were established on day 6. Primary tumors were ablated by PTT on day 10 followed by pmel ATCT on day 11. (**A**) Primary tumor growth and (**B**) primary tumor recurrence in mice treated with PTT alone or dual PTT/ATCT (n = 23 per group). *p<0.05; Z-test. (**C**) Growth of contralateral B16-F10 secondary tumors (n = 5 per group). Grey indicates tumor volume of individual mice. Red represents mean tumor volume. The data is representative of two or more experiments. *p<0.05; Student's t-test.

The immune environment established by PTT appeared beneficial to the survival and function of adoptively transferred tumor-specific T cells. Thus, we next wished to evaluate the antitumor efficacy of a dual treatment approach utilizing PTT and ATCT. Although PTT can debulk large vascularized tumors, residual tumor cells at the treatment margins may result in tumor recurrence, and the presence of tumor-specific T cells in the immediate aftermath of PTT may serve to destroy remaining cells. To evaluate tumor recurrence, B16-F10 tumors were established on contralateral flanks of mice as before. The primary tumors were treated with PTT at 5–8 mm, and half the mice also received *ex vivo*-expanded pmel T cells 24 hours later. In mice treated with PTT alone, the treated tumors recurred in 8/23 (34.8%), compared to only 3/23 (13.0%) in mice receiving dual PTT/ATCT (**Figure 7A and B**).

The combination of PTT and ATCT had equally beneficial effects on distant, untreated (contralateral) tumors. While PTT or ATCT alone were unable to inhibit the growth of pre-established B16-F10 tumors (**Figure 5D** and **7C**), dual PTT/ATCT resulted in significant inhibition of contralateral tumor growth (**Figure 7C**). These data suggest a complimentary relationship exists between PTT and ATCT. ATCT can help to prevent primary tumor recurrence after PTT, while the stimulatory effects of PTT are necessary to enhance the antitumor activity of adoptively transferred T cells for the effective treatment of distant tumor sites.

### PTT alone promotes the growth of lung metastases, but synergizes with ATCT to enhance antitumor effects

**Figure 8:**
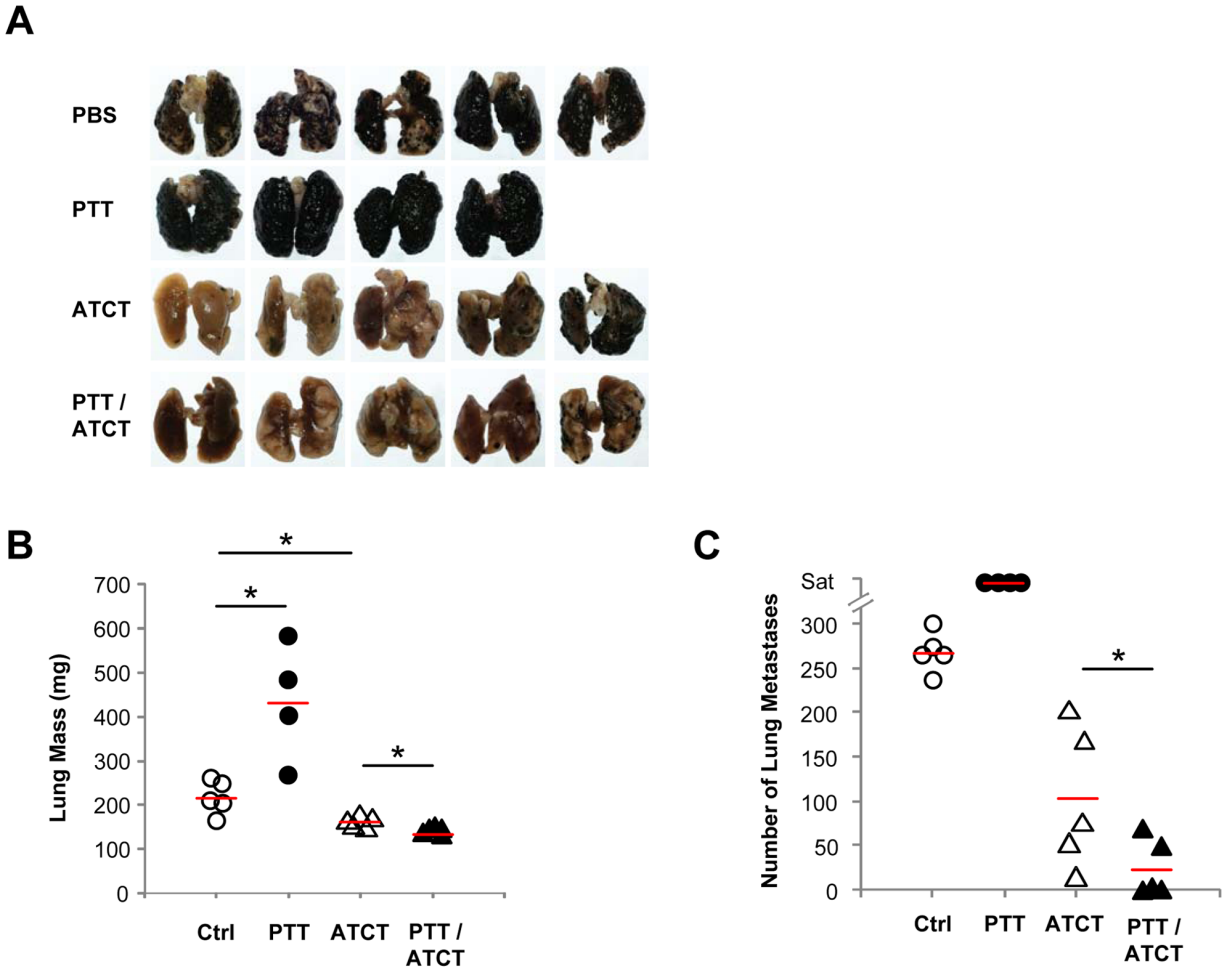
ATCT abrogates the outgrowth of lung metastases induced by PTT. B16-F10 tumors were established on day 0. Contralateral tumors were established s.c., and lung metastases were established by the administration of tumor cells i.v. on day 6. Primary tumors were ablated by PTT on day 10 followed by pmel ATCT on day 11. On day 20, mice were euthanized and lungs were harvested for analysis. (**A**) Representative lung photographs. (**B**) Lung mass and (**C**) quantification of lung metastases. Treatment groups consisted of untreated tumor bearing mice (n = 5), PTT alone (n = 4), ATCT alone (n = 5), and dual PTT/ATCT (n = 5). *p<0.05; ANOVA followed by Student's t-test with multiple comparison adjustment.

The limitations of the immune response induced by PTT are strikingly illustrated by its effects on metastatic lung disease. B16-F10 tumors were established on a single flank on day 0. On day 6, tumor cells were also administered i.v. to establish lung metastases. Primary tumors were treated with PTT on day 10, and *ex vivo* expanded pmel T cells were infused 24 hours post-ablation as before. On day 20, lungs were harvested for analysis (**Figure 8A–C**). Surprisingly, PTT greatly accelerated the growth of metastatic lung tumors in the absence of ATCT, while ATCT alone only modestly decreased lung disease burden compared to controls. However, ATCT in combination with PTT not only abrogated the enhanced metastatic tumor growth induced by PTT, but it resulted in significantly greater antitumor effects compared to ATCT alone.

## Discussion

This study highlights a dual therapeutic strategy utilizing PTT and ATCT for the treatment of metastatic cancer. Although PTT was capable of both treating local tumors and priming effector T cell responses, the inflammatory environment induced by PTT concomitantly initiated inhibitory immune mechanisms mediated by MDSC, and the resulting antitumor immune response was ineffective in eliminating distant tumors. However, we were able to exploit the stimulatory immune environment and overcome inhibitory immune components via the adoptive transfer of tumor-targeted T cells. This approach led to the eradication of both local and metastatic disease and provides a rationale for combining local “*in vivo* vaccination” by PTT and adoptive transfer of tumor-specific CTLs.

Despite clear evidence that ablative therapies can prime antitumor T cell responses, and in some cases are associated with spontaneous remission of secondary tumors [1], [2], this response is usually insufficient to eliminate metastatic disease. Such an observation is not surprising given that other immunotherapeutic strategies, such as cancer vaccines, often fail to elicit robust antitumor effects [27] because tumors evolve mechanisms to avoid immune detection and inhibit effector T cell function by establishing a suppressive tumor microenvironment consisting of Tregs and MDSC. We found that PTT monotherapy greatly increased the frequency of MDSC systemically and within contralateral tumors (**Figure 3J–L** **,** **Figure 4B**). We determined that Gr-1^High^Ly6G^+^ MDSC isolated following PTT could more potently induce T cell dysfunction (**Figure 4D**). This expansion and activation of MDSC following PTT may serve to explain why PTT monotherapy significantly worsened metastatic lung disease (**Figure 8**). Additionally, the systemic cytokine profile established in the wake of PTT could accelerate tumor cell growth by direct stimulatory effects on tumor cells. PTT was found to dramatically increase systemic levels of IL-6, which has been shown to promote the growth of B16-F10 via the activation of STAT3 signaling [28]. Both TNF-α and IL-1β, also found to be upregulated following PTT, can promote tumor growth by inducing IL-6 expression [29]. Furthermore, inflammatory cytokines may promote angiogenesis via increased secretion of VEGF and MMP9 [30].

Cytokines and chemokines released following PTT could also accelerate metastatic tumor growth by promoting MDSC generation. MDSC expansion and recruitment is observed in a wide range of acute and chronic inflammatory processes, such as cancer, burns, trauma, sepsis, and autoimmunity [31]. IL-1β promotes the expansion of MDSC [32], [33], and IL-6 acts as a downstream mediator of this process [34]. Expansion of MDSC during inflammatory processes is believed to result from increased myelopoiesis in combination with premature arrest of myeloid differentiation. Following PTT, we detected increased systemic levels of G-CSF, M-CSF, and GM-CSF, all of which are cytokines involved in promoting myelopoiesis. Furthermore, G-CSF, M-CSF, and GM-CSF have been found to accelerate tumor growth by promoting the expansion of MDSC [35]–[39]. Additionally, chemokines like CCL2 can recruit MDSC to tumor sites [40].

To generate effective antitumor results, T cell responses must be robust to overcome the suppressive tumor microenvironment. To augment the immune effects following ablative therapies, others have employed techniques to enhance the generation and effector functions of tumor-specific T cells. Toll-like receptor ligands [41], [42] and homeostatic cytokines, like IL-7 and IL-15 [43], have been employed to promote the generation and expansion of tumor-specific T cells. Alternatively, antibodies that block inhibitory T cell receptors, like CTLA-4 [26], and strategies that eliminate suppressive cell types within the tumor microenvironment, like Tregs [44], improve T cell effector functions. Although these strategies have improved the systemic antitumor effects of thermal ablation, they rely solely on the endogenous generation and expansion of tumor-specific T cells within the tumor-bearing host, a setting with an abundance of suppressive influences generated by the host immune system [45] and tumor microenvironment [46].

ATCT is an immunotherapeutic approach whereby autologous tumor-specific T cells are expanded to large numbers *ex vivo* then administered to the patient. In the setting of metastatic melanoma, ATCT can produce objective clinical responses in over 50% of patients [47], [48]. However, adoptively transferred T cells are still subject to tumor immunosuppression and often fail to persist *in vivo*, resulting in few lasting and durable responses [49]. Thus, adoptively transferred T cells are poised to benefit from the vaccine-like effect established by PTT. In the immediate aftermath of tumor ablation, pro-inflammatory cytokines and chemokines are expressed. Chemokines such as CCL2 and CCL4 could help recruit transferred T cells to the primary tumor site and thereby prevent tumor recurrence [50], [51], and inflammatory cytokines like IL-6 can promote effector T cell infiltration of distant tumor sites [52]. Furthermore, tumor-antigens are released, DC are activated, and the suppressive tumor microenvironment is destroyed. The culmination of these events could enhance the efficacy of ATCT by promoting the activation and function of transferred tumor-specific T cells. Such a combined treatment modality may help to negate the need for potentially toxic methods of T cell activation and expansion following adoptive transfer, such as host lymphodepletion or systemic IL-2 therapy. Alternatively, this therapy may further benefit from concurrent adjuvant therapies such as the use of checkpoint inhibitors like CTLA-4 antibodies [26] or via chemotherapeutic targeting of MDSC [53].

We found that the immune environment created by PTT could promote the expansion of adoptively transferred pmel T cells, leading to increased frequencies of tumor-specific T cells within the spleens and TDLN of mice (**Figure 6**). Furthermore, a combined treatment approach of PTT and ATCT had greater antitumor effects than either treatment alone. ATCT could prevent tumor recurrence following PTT, and PTT was necessary for pmel-mediated inhibition of distant tumor sites (**Figure 7**). Although a single treatment of ATCT was capable of alleviating the severity of metastatic lung disease, dual PTT/ATCT yielded the greatest antitumor effects (**Figure 8**).

This study highlights the benefit of combining PTT and ATCT for the treatment of metastatic disease and the ineffectiveness of either therapy alone. Dual PTT/ATCT greatly reduced tumor burden by eradicating primary tumors and promoting the antitumor activity of adoptively transferred T cells at distant tumor sites.

## Supporting Information

Figure S1 **HGN TEM image.** TEM image of as-synthesized HGNs. The inset shows the morphological profile of an individual HGN.
(TIF)

Figure S2 **UV-VIS-NIR.** UV-VIS-NIR spectra for as-synthesized HGNs tuned to ∼808 nm. The UV-VIS-NIR spectrum was obtained using a Cary 60 Spectrophotometer.
(TIF)
