# Supplementary figures and images for: Elimination of Metastatic Melanoma Using Gold Nanoshell-Enabled Photothermal Therapy and Adoptive T Cell Transfer
Source: PLoS One. 2013 Jul 23;8(7):e69073. doi: 10.1371/journal.pone.0069073 (PMC3720863; doi:10.1371/journal.pone.0069073)

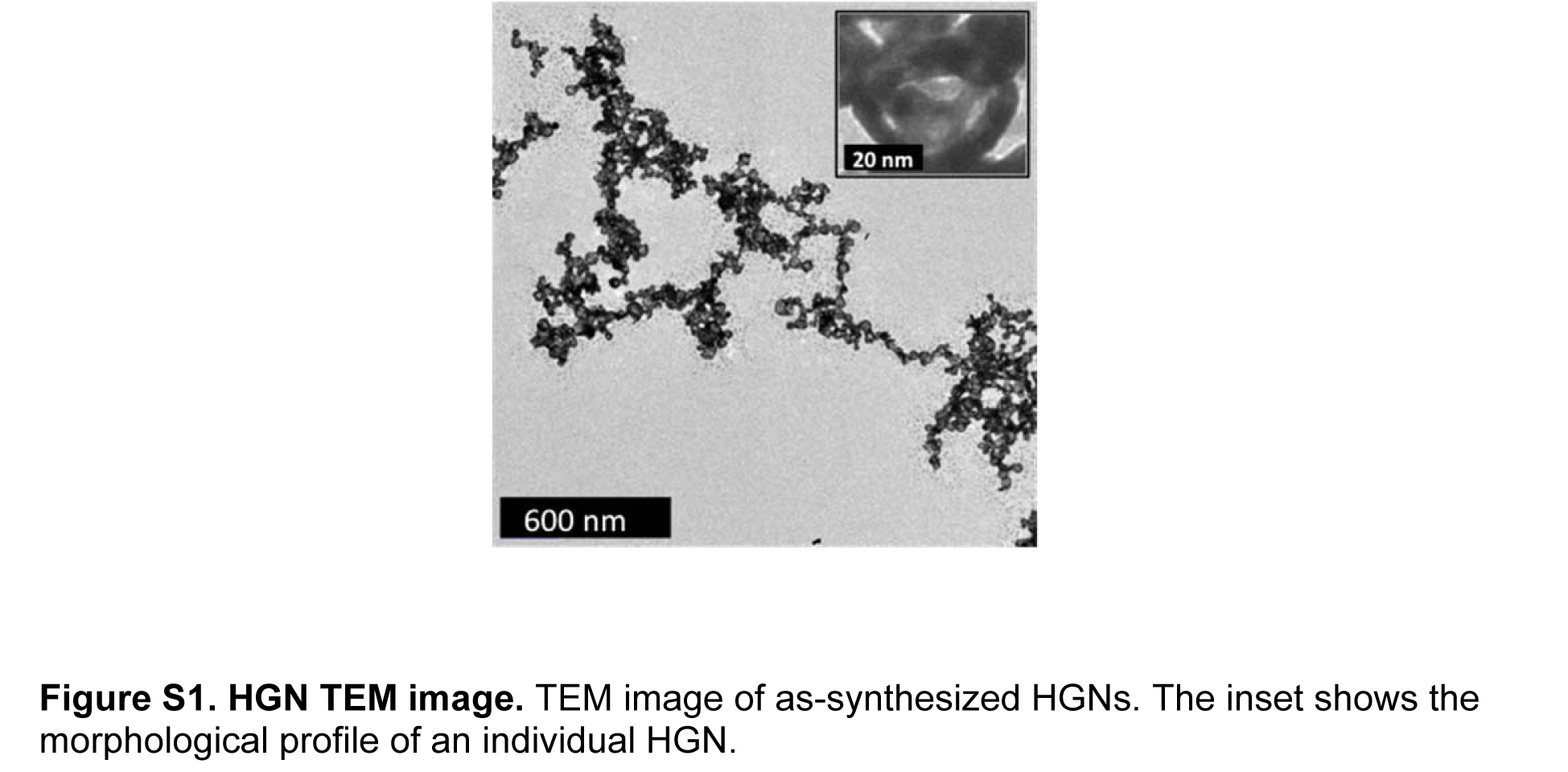

Supplement: Figure S1 — HGN TEM image. TEM image of as-synthesized HGNs. The inset shows the morphological profile of an individual HGN. (TIF) [file pone.0069073.s001.tif]

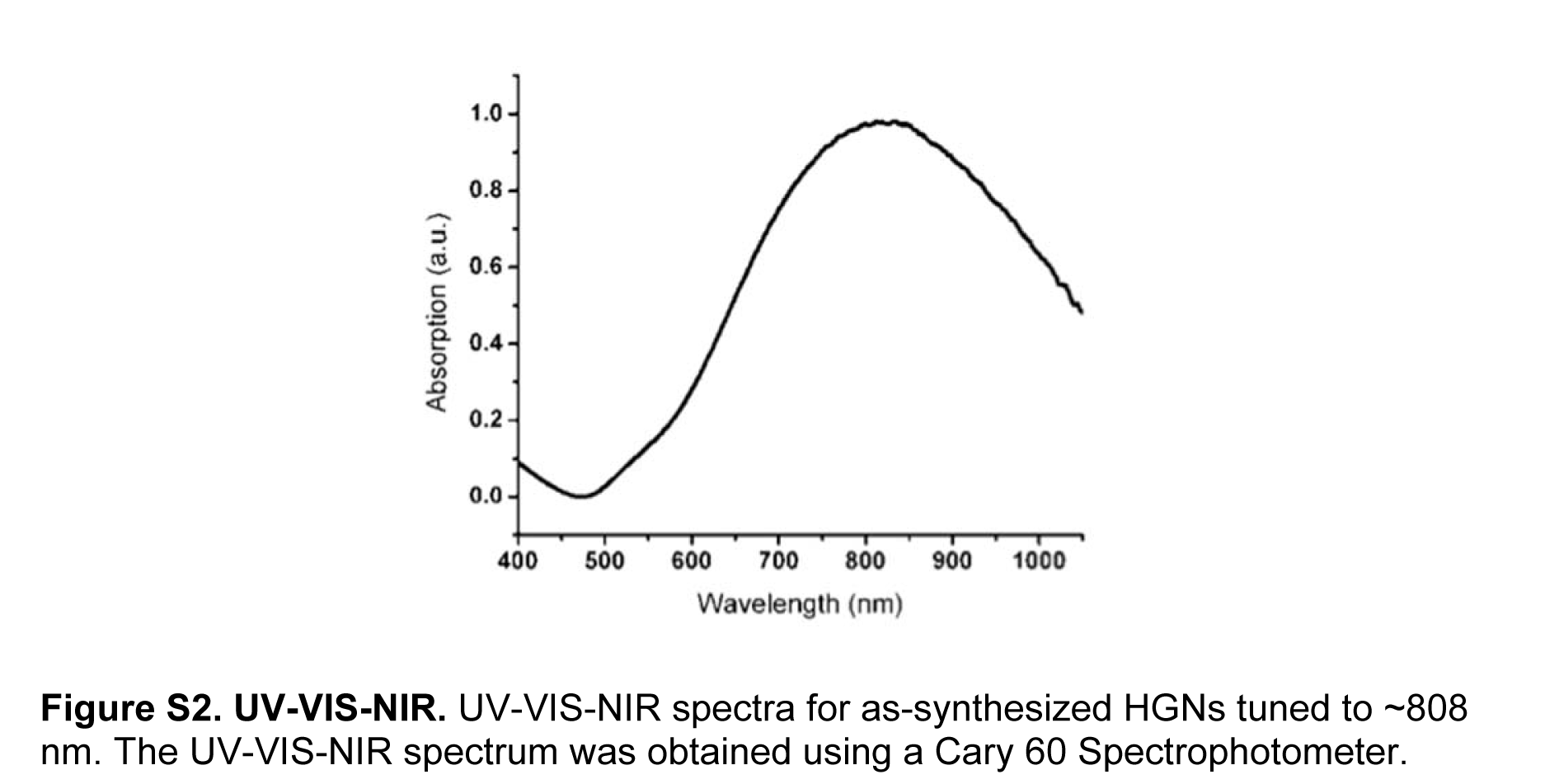

Supplement: Figure S2 — UV-VIS-NIR. UV-VIS-NIR spectra for as-synthesized HGNs tuned to ∼808 nm. The UV-VIS-NIR spectrum was obtained using a Cary 60 Spectrophotometer. (TIF) [file pone.0069073.s002.tif]
